# Preformer MOT: A transformer-based approach for multi-object tracking with global trajectory prediction

**DOI:** 10.1016/j.fmre.2024.06.015

**Published:** 2025-01-30

**Authors:** Yueying Wang, Yuhao Qing, Kaer Huang, Chuangyin Dang, Zhengtian Wu

**Affiliations:** a School of Mechanical and Electrical Engineering and Automation, Shanghai University, Shanghai 200444, China; b Lenovo, Building 1, Beijing 100085, China; c Department of Systems Engineering and Engineering Management, City University of Hong Kong, Hong Kong 999077, China; d School of Electronic and Information Engineering, Suzhou University of Science and Technology, Suzhou 215123, China

**Keywords:** Multi-object tracking, Trajectory prediction, Global association, Non-linear motion, Self-supervised learning

## Abstract

Multi-Object Tracking (MOT) is designed to accurately ascertain the positions and trajectories of moving objects within a video sequence. While prevalent methodologies primarily link detected objects across successive frames by leveraging appearance and motion attributes, some approaches incorporate implicit global correlations from multiple antecedent frames to delineate target trajectories. Nonetheless, the capability to predict trajectories over multiple future frames remains insufficiently explored, leading to a significant underutilization of pertinent information in MOT. To address this gap, we introduce a transformer-based methodology, termed Preformer MOT, which enhances the precision of nonlinear trajectory predictions in dynamic settings. This enhancement is achieved through an innovative combination of a novel motion estimation technique-trajectory prediction-and Kalman filtering. Our method not only utilizes historical trajectory data but also anticipates the future positions of the target objects up to n subsequent steps, thereby furnishing a comprehensive prediction of trajectories with extensive temporal correlations. Specifically, we develop a straightforward self-supervised trajectory prediction model that estimates the future positions of a target object based on previously observed positional data. During the correlation phase, if a trajectory disruption occurs due to overlapping, occlusion, or nonlinear movements of the detected objects, Preformer MOT is capable of making early predictions using data from multiple forthcoming frames to reestablish trajectory continuity. Empirical evaluations on pedestrian datasets such as DanceTrack and MOT17 demonstrate that our approach surpasses other contemporary state-of-the-art methods. Furthermore, Preformer MOT exhibits exceptional performance in complex marine environments, underscoring its adaptability and efficacy.

## Graphical abstract

**Figure:**
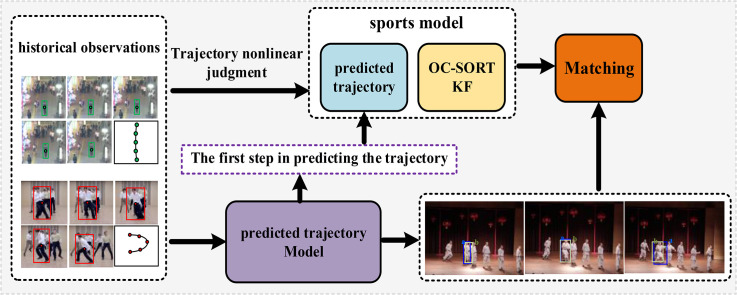

## 1 Introduction

The field of Multi-object tracking (MOT) is crucial for identifying and tracking numerous objects across video sequences, making significant contributions to traffic surveillance, autonomous vehicular technologies, and robotic vision [1], [2]. A prevalent method in this field, Tracking by Detection (TBD) [3], [4], [5], [6], divides MOT into detection and association stages. Initially, objects are detected in each frame using a detection algorithm [7], followed by the creation of motion trajectories through the association of these objects, utilizing either their appearance or motion characteristics. The Simple Online and Realtime Tracking (SORT) algorithm [8], a foundational and widely used MOT algorithm, incorporates the Kalman filter for modeling object motion. However, the Kalman filter’s assumption of linear motion is particularly challenged in complex scenarios. To overcome these challenges, a range of methods introducing advanced spatial and appearance models, along with denoising techniques, has been developed [5]. These methods aim to enhance trajectory association by combining appearance features with motion characteristics or by improving the Kalman filter with the inclusion of additional state variables, adjustments to the cost matrix, or compensation for camera motion, thus enabling more accurate predictions of non-linear motion [6], [9]. The primary goal of these developments is to enhance the robustness of Kalman filtering in situations involving non-linear motion.

In the field of computational analysis, trajectory prediction is delineated as the process of forecasting an object’s future motion trajectory by leveraging its historical motion data and current state, as explicated in seminal works [10], [11]. This prognostication is facilitated through a variety of models and algorithms within a predetermined temporal scope. It is noteworthy that trajectory prediction exhibits considerable intersection with MOT, prompting an inquiry into its limited integration within MOT endeavors. The rationale behind this limited integration can be attributed to three primary factors:

(1) Although trajectory prediction algorithms excel at forecasting the general direction of future trajectories, attaining precise accuracy at each predictive step remains a persistent challenge that requires further refinement.

(2) In high frame rate scenarios, the motion trajectories of multiple targets can often be effectively approximated through linear models, with the Kalman filter serving as a robust motion estimation tool. However, applying trajectory prediction methodologies in these situations may inadvertently reduce the precision of target association.

(3) The incorporation of advanced trajectory prediction techniques into existing tracking frameworks could entail a labor-intensive process, potentially impeding the system’s operational efficiency-a paramount consideration in real-time applications.

In this research, we present Preformer MOT, a novel methodology for MOT that leverages a straightforward self-supervised technique to predict object trajectories. By analyzing historical movement data, our approach accurately forecasts future positions, specifically targeting the next three steps to improve precision. Instead of substituting the predictions made by the Kalman filter, our trajectory forecasts serve as a complementary measure. This strategy substantially mitigates the effects of noise and error accumulation typical in the Kalman filter algorithm, adeptly handling the complexities associated with non-linear object motion.

**Fig. 1:**
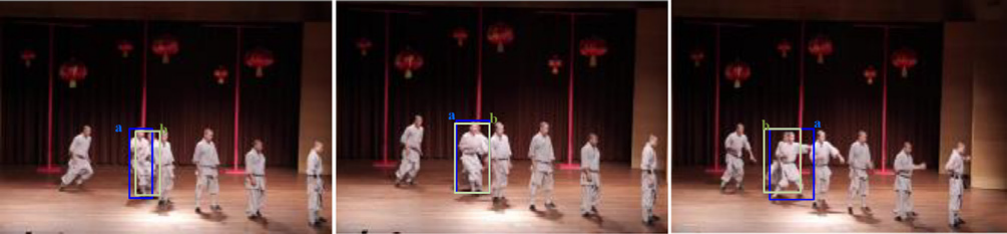
**Future Trajectory Prediction of Different Moving Targets**.

Our results demonstrate that forecasting future trajectories is pivotal for effective target association, as illustrated in Fig. 1. Specifically, if our model predicts an interaction between targets a and b in the subsequent frame, and the bounding box of target a exceeds that of target b, occlusion of target b is likely. By the third future frame, it is projected that both targets will resume their initial trajectories. Leveraging this prediction allows for the preemptive handling of occlusions upon the arrival of the second future frame, thereby efficiently mitigating issues related to object overlap, occlusion, and trajectory disruptions.

The primary contributions of our work are delineated as follows:

• We present a novel non-linear fitting approach for the motion model, which significantly mitigates estimation noise and error accumulation in intricate scenarios.
• We propose an innovative long-term prediction module that extends the positional forecasting of target objects across multiple future steps, thereby effectively capturing long-term correlational features between past and future events.
• Our algorithm demonstrates superior performance over contemporary state-of-the-art methods across two distinct datasets, highlighting its efficacy.

## 2 Related works

### 2.1 Classical tracking methods

Object tracking is a well-established research domain within computer vision, commonly addressed through two predominant methods: tracking by detection and end-to-end tracking. In the tracking by detection approach, an object detector identifies targets in each frame, which are then tracked across successive frames using motion or appearance-based similarity measures to establish their trajectories.

The SORT algorithm [8] employs the Kalman filter to predict and update object positions. DeepSORT [3] enhances the stability and robustness of object tracking by incorporating appearance similarity matching and improving cosine similarity for calculating matching scores. StrongSORT [6] enhances the feature extraction network’s performance, refines the Kalman filter algorithm, and incorporates ECC camera compensation for improved tracking accuracy. OC-SORT [5] enhances object detection capabilities, optimizes Kalman filter parameters for smoother performance, and introduces a denoising technique to effectively mitigate error accumulation. BoT-SORT [9] improves the state variables in the Kalman filter and introduces camera motion compensation to effectively handle non-linear motion. Furthermore. ByteTrack [4] optimizes the association strategy, retains low-confidence object information, and performs multiple trajectory matching, thereby achieving superior trajectory association performance. Wang et al. [12] leveraged spatio-temporal information from consecutive frames to enhance object detection accuracy and tracking performance in MOT using Spatio-Temporal maps. TransCenter [13] proposes dense multi-scale queries at the pixel level, and uses estimated target centers and sizes instead of bounding boxes to solve problems such as overlapping and occlusion of moving objects. Zhan et al. [14] employed ordinal parameters derived from studies on collective motion to model group movement patterns, addressing identity switching and performance degradation in tracking large-scale herds of animals or automated mobile robots characterized by similar appearances, frequent occlusions, and nonlinear maneuvers. FairMOT [15] estimates the target center and position on high-resolution feature maps using anchor-free object detection, and adds parallel branches to estimate pixel-level Re-ID features. Although these methods have achieved good performance, they have not made good use of global feature information.

### 2.2 Global tracking and transformer tracking

Simple feature matching between adjacent frames frequently underperforms in MOT. Several approaches adopt a global tracking strategy, utilizing information from multiple previous frames to enhance long-term tracking. For instance, Huang et al. [16] introduced GlobalTrack, a global tracker that does not assume temporal consistency of target positions and scales, enabling it to search for targets over a large area to handle potential target disappearance or tracking failure.

The blstm-mtp method [17] enhances memory updates by processing all trajectories concurrently via a multi-trajectory pooling module, which significantly improving the management of objects with similar features. GTR [18] utilizes a Transformer-based global multi-object tracking architecture to encode object features from all frames using a global tracking Transformer, followed by trajectory grouping using trajectory queries, thus achieving global trajectory tracking for all objects. Zhou et al. [19] developed a long-term target tracking algorithm that combines global tracking with temporal contextual information. Wang et al. [20] implemented a convolutional attention mechanism within a layered architecture, enhancing the mechanism by employing deformable convolutions to broaden the sensory field and capture more contextual information, and by refining the focus of attention through strategic layering. MeMOT [21] maintains a large spatiotemporal memory to store historical features of tracked objects and adaptively references and aggregates useful information as needed for trajectory tracking. However, these methods do not fully exploit the feature information from multiple past frames and merely associate long-term information with the current frame.

Several researchers have also employed Transformer structures for MOT tasks. MOTR [22] is a Transformer-based MOT framework and the first truly end-to-end multi-object tracking framework that models the long-term variation of targets by implicitly jointly learning appearance and motion changes. Tuan et al. [23] utilized historical imagery and both past and future images to track vehicles, developing graphical features and tailoring graphical similarity measures to identify vehicle objects across different cameras. MOTRV2 [24] introduces an enhanced object detector based on original MOTR original, utilizing proposal boxes from YOLOX to mitigate the performance degradation stemming from the inherent conflicts between detection and tracking tasks. Wang et al. [25] explored robust object tracking through cross-context via a Transformer architecture specifically designed for stable object tracking, with two parallel branches enhancing feature extraction. Stark [26] employs a tracking architecture centered around an encoder-decoder Transformer, where the encoder captures global spatiotemporal feature interdependencies within the target and search areas, and the decoder focuses on learning query embeddings to accurately predict the spatial positions of the target object.

Building on these concepts, our methodology also employs a Transformer structure and further advances global tracking by predicting future information from multiple frames and establishing past-future global feature associations.

### 2.3 Trajectory prediction

Trajectory prediction, a technique extensively employed in autonomous driving [27], forecasts an object’s future trajectory based on its historical data. This prediction aids autonomous vehicles in comprehending the object’s motion behavior, thereby enhancing driving safety and optimizing control strategies.

Initial studies employed Gaussian processes [28], [29] for trajectory prediction and Support Vector Machines (SVM) [30] for road condition classification. However, these models exhibited limited generalizability. Hidden Markov Models (HMMs) [31] gained prominence as a method for predicting vehicle trajectories and aiding in decision-making processes. Despite their popularity, HMMs often failed to account for interactive environmental factors, resulting in diminished predictive accuracy in complex real-world traffic situations.

The emergence of deep learning [32] has dramatically advanced the field of trajectory prediction, significantly enhancing prediction accuracy. Building on the breakthroughs in computer vision achieved through vision transformers, transformer structures [32], [33], [34], [35] have also achieved state-of-the-art results in trajectory prediction. For example, Giuliari et al. [33] demonstrated the advantages of transformers over LSTM architectures, allowing predictions to continue effectively even without new observation data. Liu et al. [35] introduced a transformer structure (mmTransformer) for multi-modal motion prediction, significantly improving object motion prediction performance. Huang et al. [36] proposed a neural prediction framework based on transformer structures for modeling the relationship between interacting agents and extracting the attention of target agents to map waypoints, predicting the behavior of other actors on the road.

Inspired by these developments, we pioneer the application of trajectory prediction in the MOT field. We contend that previous approaches have not fully leveraged the potential of historical object data, and our research concentrates on identifying and integrating long-term global correlations between past and future trajectories.

## 3 Proposed methods

**Fig. 2:**
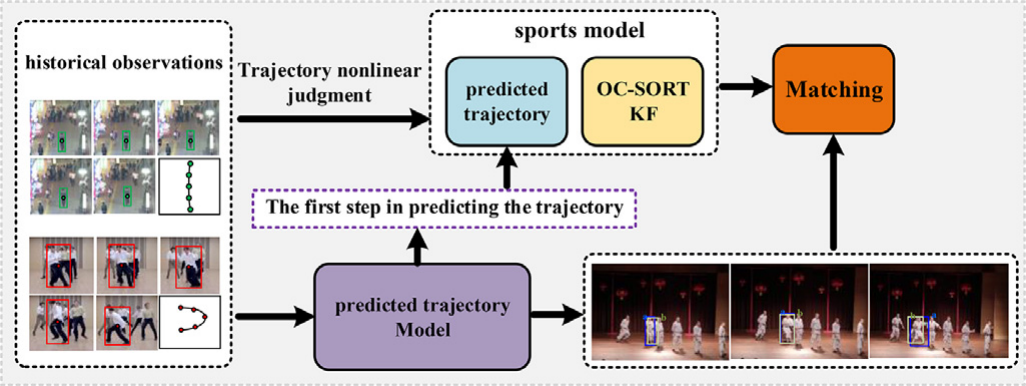
**We present a perceptron-based framework for multi-object tracking, termed Preformer MOT, which leverages historical trajectory data to derive nonlinear coefficients for the fusion of motion models**. These coefficients are then employed in trajectory prediction models, enhancing the nonlinear motion model and facilitating effective occlusion prediction.

Drawing on the strengths of deep learning pre-training frameworks and trajectory prediction techniques, we present Preformer MOT, a novel multi-object tracking approach depicted in Fig. 2. This method employs historical observational data and trajectory prediction to enhance the tracking of non-linear motion. Additionally, it predicts target positions across multiple future steps, thereby creating a detailed temporal link from past to future.

In Fig. 2’s left panel, we observe the historical trajectories of various objects, which demonstrate significant variations across different scenes, with some displaying irregular patterns. Relying solely on the Kalman filter for motion prediction may prove inadequate in these instances. To overcome this limitation, Preformer MOT employs a self-supervised learning algorithm for trajectory prediction that effectively utilizes historical data to predict future paths of objects. This technique enhances the Kalman filter’s performance, especially in complex scenarios, by improving prediction accuracy and maintaining a strong correlation.

In scenarios with high population density, significant occlusions can interrupt the continuity of object trajectories and lead to identity swaps in MOT tasks. Preformer MOT addresses these issues by extending trajectory prediction to three future steps, allowing for anticipatory predictions of potential overlaps or occlusions. This strategy significantly enhances the robustness of trajectory correlation.

Preformer MOT utilizes the Tracking by Detection (TBD) paradigm, initially employing a detector to identify objects in each frame and subsequently associating these detections with existing trajectories. To maintain comparability with other methods, the detection component adheres to standardized parameters. In the association phase, Preformer MOT innovates by integrating the trajectory prediction algorithm with the conventional motion model, thereby refining the motion prediction framework.

Section 3.1 delivers an in-depth analysis of the trajectory prediction technique through self-supervised contrastive learning. Section 3.2 explores improvements to the Kalman filter algorithm by its integration with the trajectory prediction strategy. Section 3.3 details the application of multi-step future trajectory prediction in the association mechanism.

### 3.1 Self-supervised trajectory prediction algorithm

Transformer models are proficient at capturing long-range dependencies in large datasets. However, their performance can be hindered by the availability of limited training data, often leading to suboptimal results with smaller datasets. In the field of Natural Language Processing (NLP), BERT [37] leverages a vast corpus of unlabeled text for pre-training, gaining a deep insight into language subtleties. Subsequent fine-tuning enables BERT to achieve outstanding outcomes across a variety of tasks.

In computer vision, the Masked Autoencoder (MAE) [38] adopts a technique of concealing parts of the input data during pre-training, retaining only a fraction of the features. This approach encourages the emergence of robust feature representations. Similarly, the use of pre-trained models significantly enhances task performance in downstream applications. The practice of pre-training followed by fine-tuning is proven to be effective in improving model efficacy with limited labeled data in various fields.

**Fig. 3:**
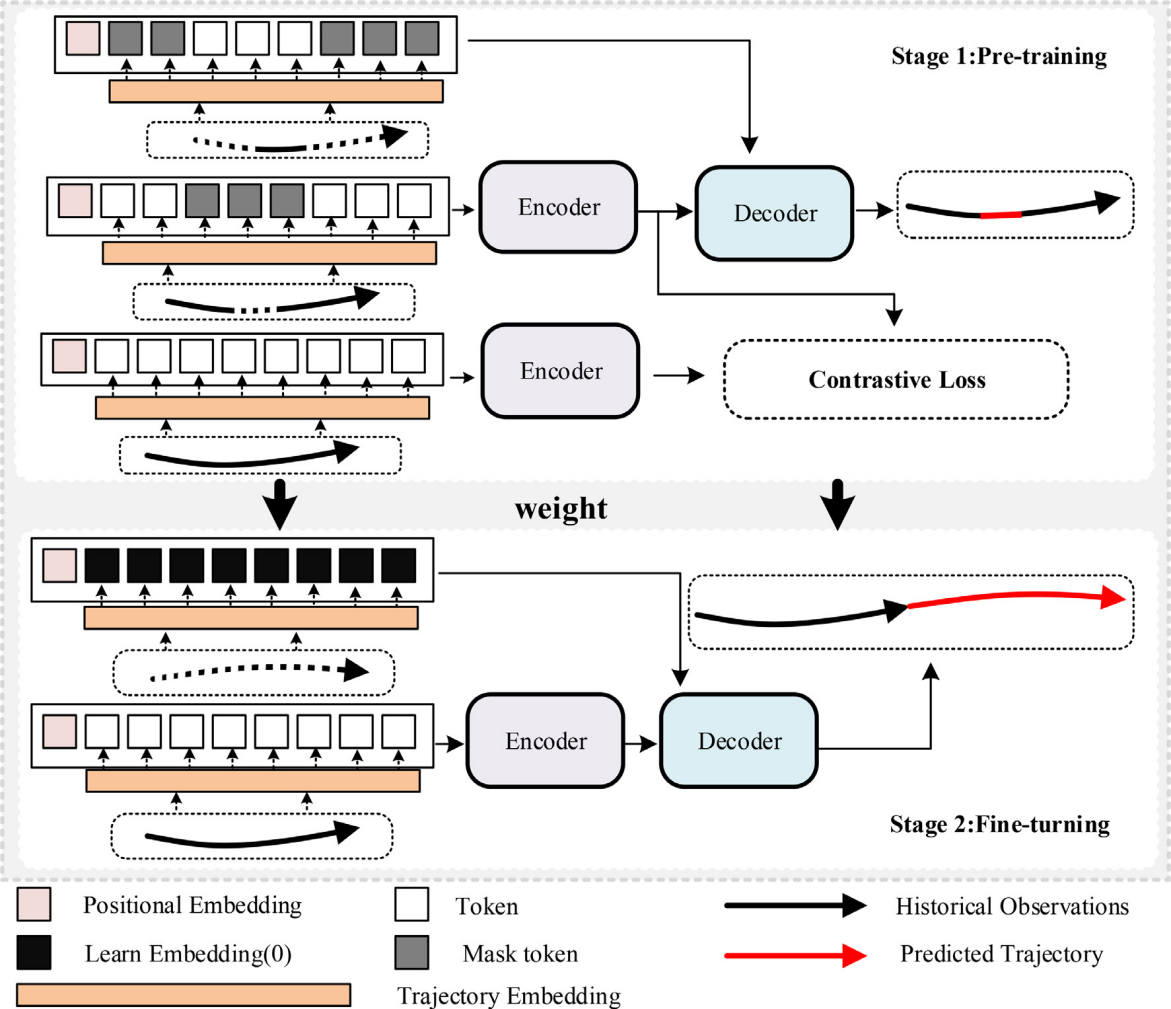
**Self-Supervised Trajectory Prediction Methods**.

In this study, we present a self-supervised transformer-based method for trajectory prediction, which is founded on contrastive learning principles. This approach incorporates a pre-training and fine-tuning framework to ensure precise predictions of future trajectories with limited data. For clarity and methodological consistency, we employ the standard encoder-decoder architecture, as illustrated in Fig. 3.

During pre-training, we solely rely on historical observational data, detailed in Section 4.1 on data preprocessing. Initially, the data is randomly masked to reduce the model’s reliance on specific input features. This masked data is processed by the encoder for feature extraction. Following this, the masked segments of the data are alternated, as depicted in the upper section of Fig. 3, allowing the model to infer missing information from the encoded features and the partially available data. This process enhances the model’s ability to interpret comprehensive data from partial inputs.

To better identify latent patterns and structures in trajectory data, we employ contrastive learning. Here, unmasked data fed into the encoder serves as the positive sample, while the feature vector from the encoding of randomly masked data acts as the negative sample. This method is designed to reveal the underlying structure and relationships between masked and unmasked data, thus enhancing the discriminative power of the features and enriching the representation for improved prediction accuracy in future tasks.

During the fine-tuning phase, we streamline the model to its essential encoder-decoder components and utilize the weights from pre-training. The preprocessed historical observational data is input into the encoder. The output from the encoder, combined with an initialized learnable zero vector, is then processed by the decoder to produce trajectory predictions.

(1) $$\begin{array}{c} O_{T}=\left\{t|1\leq t\leq 8,t\in Z\right\}\\ P_{T}=\left\{t|1\leq t\leq n,t\in Z\right\} \end{array}$$

In this equation, Z represents a positive integer, and n represents the prediction of n-step future trajectories. The indexed positions after masking can be represented as:

(2) $$O_{m}=\text{RandomMask}\left(O,T_{m}\right)$$

where RandomMask denotes random masking, and $T_{m}$ denotes random selection of m observed data points in time. The observed data after masking can be represented as

(3) $$\begin{array}{c} {\tilde{p}}_{i}=p\left(O_{m}\right) \end{array}$$

where ${\tilde{p}}_{i}$ is the observed data after occlusion. The input of the encoder can be obtained at this time as

(4) $${\mathrm{Enc}}_{\text{m}}=\text{Pos}+\text{linear}\left({\tilde{p}}_{i}\right)$$

where Pos denotes the sine and cosine position encoding and linear represents the linear mapping layer. The decoder can reconstruct the masked trajectory data. Its input has two parts, one part comes from the output of the encoder. In the pre-training stage, the other part comes from the local observation value, which can be expressed as

(5) $${\mathrm{Dec}}_{\text{pre}}=\left[{\mathrm{Enc}}_{\text{m}},p\left(O-O_{m}\right)\right]$$

Finally, only the mean squared error between predicted values for occluded areas and historical observations is computed:

(6) $${\mathrm{Loss}}_{\text{pre}}=\text{MSE}\left({\mathrm{Dec}}_{\text{pre}},p\left(O-O_{m}\right)\right)$$

Contrastive learning, recognized for its effectiveness in pre-training neural networks, refines representations by promoting similarity within identical samples and enforcing distinctiveness among different samples in latent space. Within the domain of trajectory prediction, we leverage contrastive learning to distinguish between real and masked data after encoding, facilitating the reconstruction of masked trajectories. This process involves the integration of pre-masked data with positional encoding through an encoder module, where the output from the original data acts as the positive sample, and the output from the masked data serves as the negative sample. The loss is calculated using the Negative Cosine Similarity (NCS) [39] function, expressed as follows:

(7) $${\mathrm{Loss}}_{\text{cl}}=\text{NCS}\left({\mathrm{Enc}}_{\text{m}},\mathrm{Pos}+p\left(O\right)\right)$$

Finally, the total loss in the pre-training phase is given by:

(8) $$\mathrm{Loss}={\mathrm{Loss}}_{\text{pre}}+{\mathrm{Loss}}_{\text{cl}}$$

In the fine-tuning stage, we load the weights of the pre-training stage, at this time the input of the encoder is the complete observation track,

(9) Enc=Pos+linear(p(OT))

where linear represents the linear mapping layer. At this time, the input and output of the decoder are respectively

(10) Dec=[Enc,LE(0)]

where LE(0) is a learnable vector initialized with all 0s, and the loss function at this time is:

(11) Loss=MSE(Dec,p(PT))

### 3.2 Nonlinear motion model

In conventional motion models, the Kalman filter is employed to forecast the future motion characteristics of an object based on its initial detections. However, its assumption of linear and constant velocity motion significantly limits its accuracy in predicting nonlinear trajectories. To overcome this limitation, we have refined the Kalilman filter by incorporating a trajectory prediction method that utilizes spatial data from multiple prior observations to enhance the accuracy of future position predictions, thus improving the model’s capacity to manage nonlinear movements.

Furthermore, we introduce a memory function that stores the centroid coordinates from each object’s past observations. Should the number of past observations exceed eight, the oldest is removed, maintaining a repository of only the most recent eight observations. Employing the trajectory prediction model described in Section 3.1, we are able to accurately project future trajectories.

It is crucial to recognize that for objects with fewer than eight observations, the Kalman filter is still utilized for predicting immediate future motion, ensuring the integrity of the motion model. This approach guarantees that a prediction is available for each motion step in every trajectory. The predictions generated by the trajectory prediction model are represented as $V_{\text{pre}}$, which has proven to be effective in predicting the nonlinear movements of objects.

Theoretically, the predictions from $V_{\text{pre}}$ could potentially replace those made by the Kalman filter. However, in practice, scenarios often involve a combination of both linear and nonlinear trajectories, making it impractical to rely solely on $V_{\text{pre}}$ for linear motion predictions. To address this issue, we introduce a nonlinear coefficient that enhances the predictions made by the Kalman filter.

(12) $$V_{F}=\alpha \times V_{\text{pre}}+\left(1-\alpha \right)\times V_{\text{kalman}}$$

In these equations, $V_{\text{kalman}}$ symbolizes the motion model derived from the Kalman filter, while $V_{F}$ signifies the enhanced motion model that includes nonlinear adjustments. The coefficient α, which varies between 0 and 1, quantifies the nonlinearity of each trajectory, with values approaching 1 indicating greater nonlinearity. The methodology for computing α is detailed in Algorithm 1.



In this context, Unit_V is defined as the unit vector, while ${\text{DisP}\_V}_{t}$ denotes the displacement vector. The term $POS_{t}$ refers to the projection of the displacement vector onto a line, and ${\text{POS}}_{t}^{\prime }$ restricts this projection to ensure it remains within the start and end points. ${\text{Pos}\_D}_{t}$ is the perpendicular distance from each displacement vector to the line, ${\text{Pos}\_\text{unit}\_D}_{t}$ represents the corresponding unit vector, and avg_P_D signifies the average perpendicular distance across trajectories. We calculate the nonlinear coefficient nonlin, which is subsequently normalized to yield the final normalized nonlinear coefficient nonlin_norm. The notation ∥∥ indicates vector magnitude, and · symbolizes the dot product. Utilizing these components, we have formulated a motion model to estimate nonlinear trajectories in complex environments.

In Algorithm 1, the historical position data of the target is inputted to assess the nonlinearity of each trajectory by examining the curve complexity of various historical trajectories of the target. This assessment is quantified on a scale from 0 to 1, resulting in the final non-linearity coefficient α.

In environments characterized by high population density and significant visual obstructions, we enhance the robustness of trajectory association by incorporating predictions based on multi-step future information. Algorithm 2 details our method for quantifying interactions among multiple trajectories, aimed at identifying and quantifying such interactions within a system.

Initially, two empty lists are initialized: I to log interactions between trajectories, and C to track the closest trajectory for each individual trajectory. The algorithm checks for the presence of trajectories within the system and, upon detection, computes the Euclidean distance between each pair of trajectories at every step. These distances are stored in a matrix D, and the corresponding trajectory indices and step lengths are recorded.

The algorithm utilizes a matrix L to store the latest data for each trajectory, with dimensions corresponding to the number of trajectories N and the sequence of steps S. The output list I identifies potential occlusions by noting the trajectory ID, the specific step, and the distance between the midpoints of the two trajectories at that step. A threshold $I_{t}$ defines the critical distance below which an occlusion risk is considered significant, indicating that shorter distances increase the likelihood of occlusion. Each interaction is characterized by the indices of the two interacting trajectories, the step number, and the distance at that step. The list C maintains a record of the nearest trajectory to each one, with D representing the matrix of distances, s denoting the current step, and d indicating the distance between trajectories at step s. Ultimately, the algorithm outputs the lists I and C, representing interactions and nearest trajectories, respectively.

The algorithm iteratively evaluates each step for every pair of trajectories. If the distance between any two trajectories during an iteration falls below a predefined interaction threshold, the algorithm records this interaction. These interactions are stored in a list, formatted as dictionaries with keys representing the interacting trajectories, the interaction step, and the distance between them. After cataloging all interactions, the algorithm identifies the nearest trajectory for each by summing the distances between every pair of trajectories across all steps and selecting the trajectory with the smallest total distance. To mitigate the impact of potential future occlusions, the algorithm records the indices of trajectories and the number of steps where occlusions are likely. Given the decrease in accuracy for predicting future positions with an increasing number of forecasted steps, the algorithm limits its focus to interactions among distinct trajectories for the next three steps. Furthermore, it identifies the nearest trajectory for each, a crucial detail for the subsequent phase of trajectory association.

### 3.3 Association

In the preceding section, we introduced a non-linear motion prediction model that proactively addresses potential occlusions. This model, based on OCsort, relies solely on non-linear trajectory estimations for predicting motion, deliberately excluding appearance models. To enhance the accuracy of trajectory evaluation and association, particularly in scenarios where initial associations fail, we have incorporated positional interaction data from future n steps.

A detailed analysis is conducted at the second future step to detect potential occlusions. If occlusions are identified and resolved by the third future step, we record the IDs of trajectories that are at risk. In subsequent trajectory updates, we revisit previously unassociated trajectories, specifically looking for IDs predicted to be occluded. Upon locating such IDs, we verify if the number of detection boxes corresponds with the expected number of trajectories. A discrepancy, particularly a decrease in detection boxes, indicates an occlusion at that frame, which likely caused the failure in detection and, by extension, association. To rectify this, we implement an additional association step where the occluded object’s bounding box is replaced with a projection from the non-linear trajectory estimation and reassociated to maintain the continuity of its original trajectory.

## 4 Experimental

### 4.1 Datasets and metrics

**Datasets**: Our trajectory prediction model, designed for multi-object tracking, utilized a refined version of the Dancetrack dataset’s ground truth for training [40]. This dataset was partitioned into distinct training and testing sets. Adhering to the conventional structure of trajectory prediction datasets, the first six columns of the Dancetrack dataset’s ground truth file were selected. We converted the bounding box data from columns three to six into centroid coordinates (x, y) and scaled the first column’s values by a factor of 10. Following this, the dataset was organized based on the ascending values of the first column, resulting in a streamlined four-column dataset. This dataset comprises the frame rate (first column), object ID (second column), and the object’s centroid coordinates (third and fourth columns). Notably, the Dancetrack dataset includes cases of object occlusion, where annotations for occluded objects are temporarily absent. However, upon the resolution of occlusion, the trajectory ID for the occluded object is restored. Such interruptions in trajectory data pose challenges to accurate prediction. To address this, we approximated the position of an occluded object by averaging its positions from the frames immediately preceding and succeeding the occlusion.

For the multi-object tracking stage, we assessed our method using the MOT17 [41] and Dancetrack [40] datasets. Both datasets are designed for pedestrian tracking, but they differ in the nature of the motion targets: MOT17 primarily includes linear motion targets, while Dancetrack features predominantly non-linear motion targets with frequent and severe occlusions.

We conducted a further evaluation of Preformer MOT’s multi-target tracking capabilities in complex maritime environments. For this purpose, we employed a dataset specifically designed for sea surface multi-target tracking, provided by the 716th Research Institute of the China State Shipbuilding Corporation. This dataset consists of high-definition video sequences recorded from unmanned boats, covering four distinct maritime scenarios: port area, port departure, port entry, and open sea. It features annotations for common sea surface targets across these scenarios. Comprising 38 video sequences, with each sequence containing 300 to 1500 frames, the dataset poses significant challenges, including unbalanced target categories, target occlusion, small target sizes, and difficulties in target association amidst complex lighting and carrier maneuvers. We divided the dataset into a training set with 30 sequences and a test set with 8 sequences to conduct our evaluation.

**Metrics**:We utilized the Higher Order Tracking Accuracy (HOTA) [42] as the primary metric for evaluating our method. In addition, we placed emphasis on assessing detection accuracy (DetA), association accuracy (AssA), Multiple Object Tracking Accuracy (MOTA), and the IDF1 metric [43], [44].

**Implementation Details**:Our implementation is built upon OC-sort. To maintain a fair comparison, we adopted the object detection from the existing baseline. Specifically, we employed the YOLOX [45] detector, using the same weights as bytetrack [46]. The fundamental parameter settings were preserved consistent with OC-sort, setting a detection confidence threshold at 0.6 and an association confidence threshold at 0.3.

### 4.2 Benchmarks evaluation

We assessed the performance of Preformer MOT relative to other leading methods on the MOT17 and DanceTrack datasets. These methods include FairMOT [47], GRTU [50], TransCenter [48], TransTrack [49], TransMOT [53], MOTR [52], QDtrack [51], ByteTrack [46], OC-SORT [5], C-BIOU [54] and MotionTrack [57]. The results of these experiments are presented in Table 1 and Table 2.

**Table 1:** **Results on MOT17-test with the private detections. Methods in the blue blocks share the same detections. The best of these results are bolded**.

Tracker	HOTA↑	MOTA↑	IDF1↑	FP(104)↓	FN(104)↓	IDs↓	Frag↓	AssA↑	AssR↑
FairMOT ([47])	59.3	73.7	72.3	2.75	11.7	3303	8,073	58.0	63.6
TransCt ([48])	54.5	73.2	62.2	2.31	12.4	4614	9,519	49.7	54.2
TransTrk ([49])	54.1	75.2	63.5	5.02	**8.64**	3603	4,872	47.9	57.1
GRTU ([50])	62.0	74.9	75.0	3.20	10.8	1812	**1,824**	62.1	65.8
QDTrack ([51])	53.9	68.7	66.3	2.66	14.66	3378	8,091	52.7	57.2
MOTR ([52])	57.2	71.9	68.4	2.11	13.6	2,115	3897	55.8	59.2
TransMOT ([53])	61.7	76.7	75.1	3.62	9.32	2,346	7719	59.9	66.5
ByteTrack ([46])	63.1	80.3	77.3	2.55	8.37	2196	2,277	62.0	68.2
OC-SORT ([5])	63.2	78.0	77.5	**1.51**	10.8	1950	2,040	63.2	67.5
C-BIOU ([54])	64.1	**81.1**	79.7	2.38	10.17	1640	2034	63.7	68.1
Preformer MOT	**64.2**	79.1	**79.9**	1.83	9.82	**1248**	1974	**64.0**	**69.6**

**Table 2:** **Results on DanceTrack test set. Methods in the blue blocks share the same detections. The best of these results are bolded**.

Tracker	HOTA↑	DetA↑	AssA↑	MOTA↑	IDF1↑
Center Track ([55])	41.8	78.1	22.6	86.8	35.7
FairMOT ([47])	39.7	66.7	23.8	82.2	40.8
QDTrack ([51])	45.7	72.1	29.2	83.0	44.8
TransTrk ([49])	45.5	75.9	27.5	88.4	45.2
TraDes ([56])	43.3	74.5	25.4	86.2	41.2
MOTR ([52])	54.2	73.5	40.2	79.7	51.5
SORT ([8])	47.9	72.0	31.2	91.8	50.8
DeepSORT ([3])	45.6	71.0	29.7	87.8	47.9
ByteTrack ([46])	47.3	71.6	31.4	89.5	52.5
MotionTrack ([57])	52.9	80.9	34.7	91.3	53.8
OC-SORT ([5])	55.1	80.3	38.3	**92.0**	54.6
Preformer MOT	**59.0**	**82.1**	**42.6**	91.5	**57.8**

**MOT17** [41]: The experimental results on the MOT17 dataset are presented in Table 1. Preformer MOT outperformed other methods, achieving the highest HOTA and IDF1 scores (64.2 HOTA and 79.9 IDF1). It exhibited improvements in all metrics compared to the second-best method. However, the overall performance improvement was limited compared to the results on the DanceTrack dataset. This is due to the fact that a significant number of trajectories in the MOT17 dataset can be approximated as linear motion, limiting the complementarity of the non-linear motion model in Preformer MOT. Nonetheless, Preformer MOT still demonstrated an overall performance improvement on the MOT17 dataset, underscoring the superiority of our method.

**Fig. 4:**
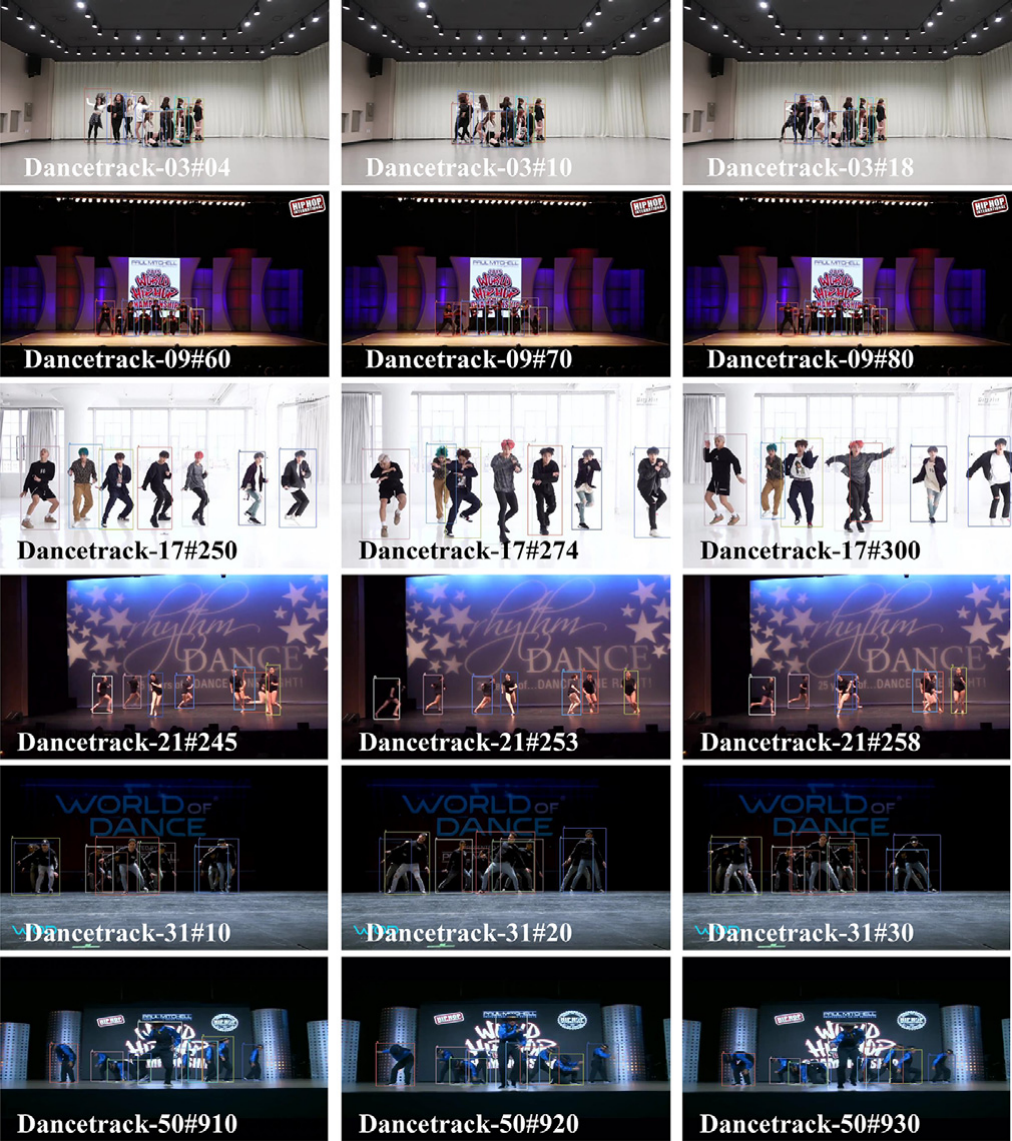
**Visualization of Preformer MOT Performance on the MOT17 Dataset**.

Fig. 4 showcases a subset of visualization results from the proposed method applied to the MOT17 dataset. Each row represents tracking results from different frames within the same video sequence, demonstrating the robust performance of Preformer MOT across various scenarios. As evident in rows 1 and 3, Preformer MOT maintains effective tracking in denser scenes. Rows 2 and 4 feature numerous occluded targets, yet these are still accurately detected. This is attributed to our method’s ability to predict multi-step future trajectories. Even when targets are lost due to occlusion, they are re-associated along the predicted trajectory upon reappearance, thereby enhancing tracking results through the incorporation of future prediction information.

**Dancetrack** [40]: Table 2 displays the experimental results on the Dancetrack test set. Using only the motion model, Preformer MOT achieved a HOTA score of 59.0, surpassing all comparison methods and demonstrating a significant advantage. In addition to these results, we achieved optimal performance in both detection accuracy and association accuracy metrics. Specifically, our correlation accuracy improved by approximately 6%. These results further substantiate that Preformer MOT effectively supplements non-linear motion models and successfully tackles Dancetrack’s challenges, such as numerous non-linear motion trajectories and severe occlusions.

**Fig. 5:**
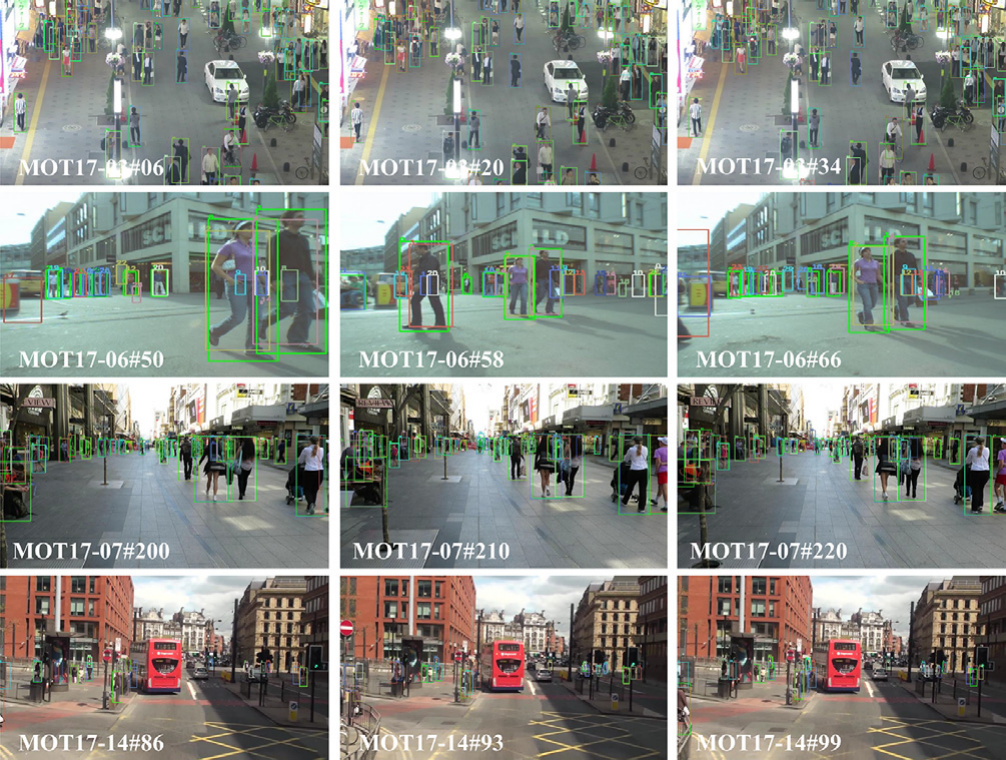
**Visualization of Preformer MOT Performance on the Dancetrack Dataset**.

Fig. 5 presents a selection of visualization results from the proposed method applied to the Dancetrack dataset. Each row corresponds to the tracking outcomes from different frames within the same video sequence. Given the dataset’s complexity, characterized by a high degree of nonlinear motion and highly similar target objects, conventional MOT methods struggle to perform effectively. However, the proposed Preformer MOT, which employs a trajectory prediction method for motion estimation, excels in fitting nonlinear motion. It predicts future multi-step motion positions, thereby theoretically establishing a continuous trajectory. This approach effectively addresses the issue of occlusion, as our association strategy correctly re-associates the target upon reappearance.

**Fig. 6:**
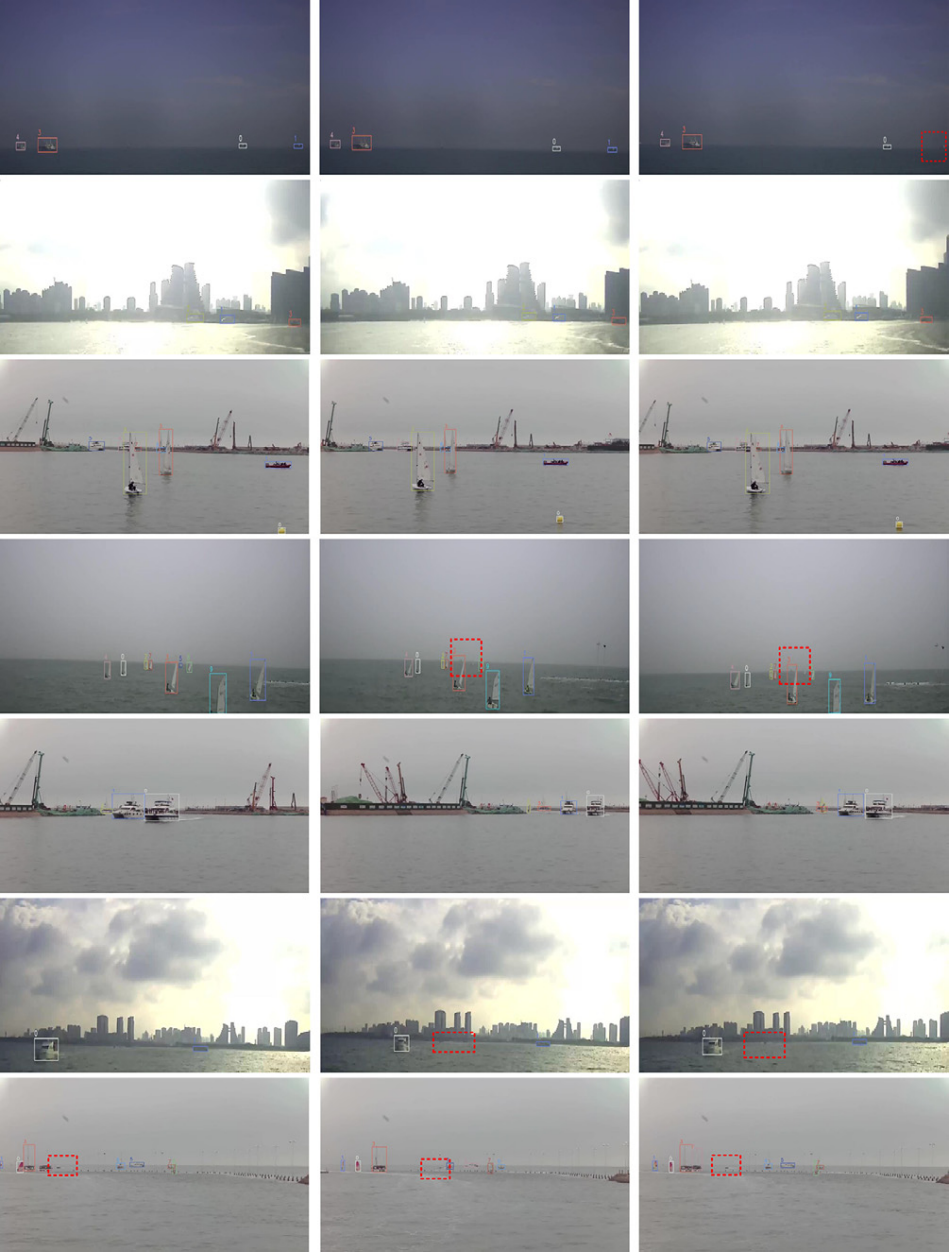
**Depiction of Preformer MOT Application on the Sea Surface Multi-Target Tracking Dataset**.

Fig. 6 displays a selection of visualization results from the proposed method applied to a sea surface multi-target tracking dataset. Each row represents the visualization outcomes from different sea surface scenarios. The red dotted line encloses areas where targets exist but have not been detected. As seen in rows 1 and 2, Preformer MOT effectively tracks all targets when detection is accurate. Rows 4 and 7 demonstrate that Preformer MOT handles occlusion and small target tracking exceptionally well. In rows 6, where the target object moves non-linearly, Preformer MOT continues to exhibit strong tracking performance.

### 4.3 Ablation studies

In this section, we scrutinized various components of Preformer MOT and affirmed the efficacy of the proposed methods through experiments conducted on the DanceTrack dataset. We carried out three sets of experiments. The first set utilized the Kalman filtering algorithm as the motion model (Kalman). The second set enhanced the motion model with a non-linear trajectory prediction algorithm (NMTS). In the third set, we further integrated multi-step trajectory prediction (PFT). The experimental results are presented in Table 3.

**Table 3:** **Ablation studies performed on the dancetrack dataset. The best of these results are bolded**.

kalman	NMTS	PFT	HOTA↑	DetA↑	AssA↑	MOTA↑	IDF1↑
✓			55.1	80.4	40.4	91.2	54.9
✓	✓		58.4	81.7	41.6	91.4	56.8
✓	✓	✓	**59.0**	**82.1**	**42.6**	**91.5**	**57.8**

After enhancing the motion model with a nonlinear approach, we observed a notable improvement in association performance on the DanceTrack dataset. Specifically, the improvements were 3.3 points for HOTA (from 55.1 to 58.4) and 1.2 points for AssA (from 40.4 to 41.6), effectively addressing the challenges associated with nonlinear motion. Although the prediction of future multi-step trajectories somewhat mitigated the occlusion problem, the overall performance improvement was relatively limited. This limitation primarily stems from the need for further refinement in the accuracy of trajectory prediction methods.

The trajectory prediction method employed in this study serves as a straightforward yet effective baseline. Future work aimed at improving the accuracy of trajectory prediction can further enhance the performance of Preformer MOT.

## 5 Conclusion

In this study, we delved into the application of trajectory prediction methods within the realm of MOT. Preformer MOT capitalizes on historical trajectory data to extend the prediction of object positions up to three future steps, thereby generating anticipated trajectories for all objects. This approach facilitates early mitigation of potential challenges such as overlap and occlusion. Moreover, the non-linear motion prediction of trajectories serves as an effective supplement to the linear motion prediction of Kalman filtering, thereby bolstering the robustness of the motion model. The trajectory prediction component of Preformer MOT employs a straightforward yet potent baseline. The incorporation of more advanced trajectory prediction methods could potentially further augment the performance of MOT tasks. We anticipate that Preformer MOT will make a significant contribution to the progression of the field of Multiple Object Tracking.

Although the Preformer MOT achieves commendable performance in multi-target tracking, its trajectory prediction model, which necessitates additional training, lacks simplicity and elegance. In future work, we aim to further explore end-to-end multi-target tracking methods that incorporate trajectory prediction.

## Declaration of competing interest

The authors declare that they have no conflicts of interest in this work.
